# SNDR Limits of Oscillator-Based Sensor Readout Circuits

**DOI:** 10.3390/s18020445

**Published:** 2018-02-03

**Authors:** Fernando Cardes, Andres Quintero, Eric Gutierrez, Cesare Buffa, Andreas Wiesbauer, Luis Hernandez

**Affiliations:** 1 Department of Electronics Technology, Carlos III University of Madrid, 28911 Leganes, Spain; anquinte@ing.uc3m.es (A.Q.); egutie1@ing.uc3m.es (E.G.); luish@ing.uc3m.es (L.H.); 2 Infineon Technologies Austria AG, Villach 9500, Austria; Cesare.Buffa@infineon.com (C.B.); Andreas.Wiesbauer@infineon.com (A.W.)

**Keywords:** oscillator-based sensor, phase noise, Sigma-Delta modulation, time-domain circuits, VCO-ADC

## Abstract

This paper analyzes the influence of phase noise and distortion on the performance of oscillator-based sensor data acquisition systems. Circuit noise inherent to the oscillator circuit manifests as phase noise and limits the SNR. Moreover, oscillator nonlinearity generates distortion for large input signals. Phase noise analysis of oscillators is well known in the literature, but the relationship between phase noise and the SNR of an oscillator-based sensor is not straightforward. This paper proposes a model to estimate the influence of phase noise in the performance of an oscillator-based system by reflecting the phase noise to the oscillator input. The proposed model is based on periodic steady-state analysis tools to predict the SNR of the oscillator. The accuracy of this model has been validated by both simulation and experiment in a 130 nm CMOS prototype. We also propose a method to estimate the SNDR and the dynamic range of an oscillator-based readout circuit that improves by more than one order of magnitude the simulation time compared to standard time domain simulations. This speed up enables the optimization and verification of this kind of systems with iterative algorithms.

## 1. Introduction

**Figure 1:**
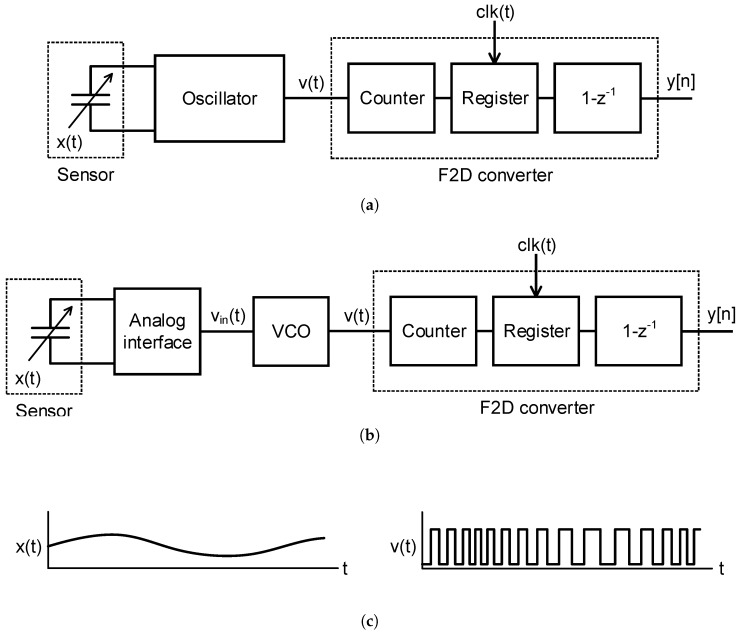
Example of two oscillator-based sensor readout circuits. The sensing element can be integrated into the oscillator (**a**) or can be connected to an analog interface that generates an intermediate signal $v_{in}$ that modulates the oscillator (**b**). In both cases, the input measurand x(t) modulates the frequency of the oscillation v(t) (**c**).

Time-domain encoding has gained popularity due to the challenges that low supply voltages suppose to conventional analog circuits in deep submicrometer processes [1,2,3,4,5]. Time-encoding systems benefit from technology scaling by having higher resolution in time and smaller area. One of the most extended time-encoding systems is based on frequency modulation (FM) of an oscillator. This approach has been used during the last years to build efficient time-domain sensor readout circuits [6,7,8,9,10,11,12,13,14]. Figure 1 provides two examples of oscillator-based sensor readout systems. Figure 1a depicts a sensor readout circuit on which a sensing element (a capacitor in this example) is part of an oscillator. The sensor can be either a capacitive, inductive or resistive element that changes the frequency by affecting the resonance frequency or time constant of the oscillator. The measurand (x(t)) modulates the frequency of the oscillation (v(t)), which can be processed by a frequency-to-digital (F2D) converter whose output is a digital sequence proportional to the oscillation frequency. Alternatively to this sensor-controlled oscillator, the sensor can be connected to an analog interface circuit that generates a voltage ($v_{in}$) which drives a voltage-controlled oscillator (VCO). The output of the VCO (v(t)) is also processed by a F2D converter to produce a digital sequence, as shown in Figure 1b. In both cases, the measurand x(t) is encoded in the frequency in the oscillator output v(t) (see Figure 1c). The combination of an oscillator and the F2D converter of Figure 1 works as a continuous-time first-order ΣΔ modulator (CTΣΔM), which shows first-order noise-shaping [15,16].

**Figure 2:**
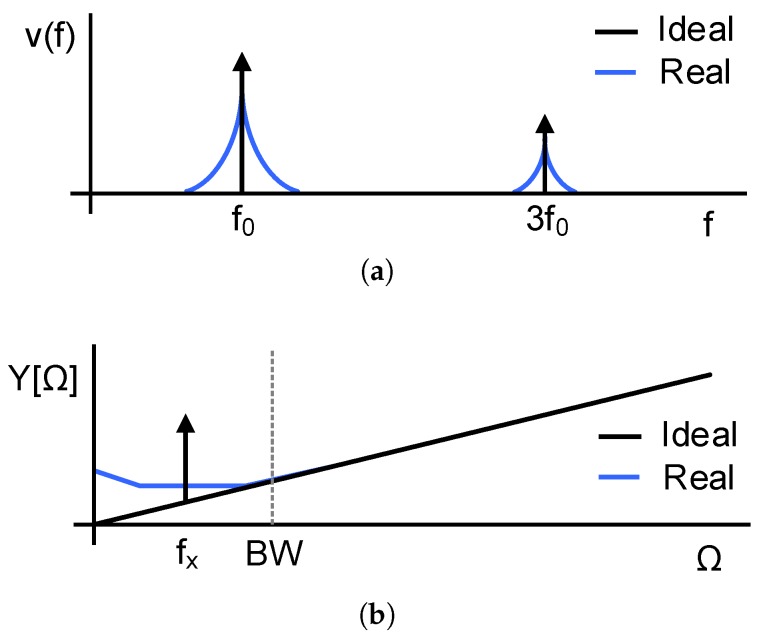
Effects of noise in the spectra of a VCO-ADC. (**a**) Spectrum of v(t); (**b**) Spectrum of the output of the converter y[n] assuming a sinusoidal input.

Ideally, the oscillation v(t) should be a square signal whose frequency is determined by the measurand and circuit parameters, and its spectrum at rest would be composed by Dirac deltas at the oscillation frequency and its harmonics, as shown in Figure 2a. In this case, the output of the modulator y[n] would only contain the quantization noise produced by the F2D converter, and an input signal if any applied. However, circuit noise is unavoidable and produces random variations in the oscillation frequency, which is also described as phase noise and appears in the spectrum of v(t) around the center frequency ($f_{0}$) and its harmonics. These random fluctuations are indistinguishable from frequency variations produced by the measurand, and therefore they establish a limit in the accuracy of the sensor regardless quantization noise. Phase noise is downconverted by the F2D converter and it is reflected in the output sequence as a low frequency noise, as shown in Figure 2b assuming a sinusoidal input. Consequently, the power error resulting from integrating the noise PSD inside the band of interest increases, limiting the resolution of the readout circuit.

Phase noise theory has been studied in numerous works, like Leeson`s model [17,18] and many others [19,20,21,22]. Phase noise calculations are complex due to the time-varying nature of oscillators and the variety of topologies existing. Hajimiri et al. [23] introduced a function called “impulse sensitivity function” (also known as ISF and Γ(x)) useful to describe how the phase fluctuates when the oscillator is disturbed by impulses at different instants along the oscillation. Nowadays, designers have available software tools capable of accurately simulating the phase noise of complex oscillator circuits.

**Figure 3:**
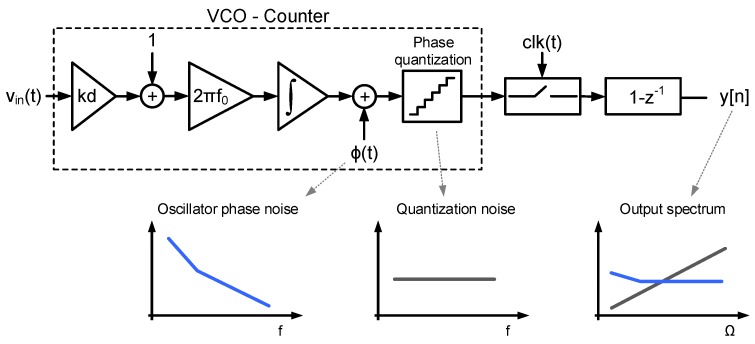
Classical approach to estimate the influence of phase noise in the performance of first-order VCO-ADCs.

On the other hand, the influence of phase noise in the performance of certain oscillator-based data acquisition systems has been studied during the last years [9,15,24,25]. These works focus on first-order noise-shaping VCO-ADC architectures like the ones depicted in Figure 1, which can be modeled as shown in Figure 3. A VCO followed by a digital counter operates as a frequency integrator whose output is the oscillator phase quantized in discrete steps. The gain $k_{d}$ represents the oscillator sensitivity, which will be described in details in Section 3. Phase quantization implies the addition of a quantization noise signal, which in most of the cases can be assumed random and independent from phase fluctuation due to circuit noise (ϕ(t)). Both noise signals are sampled and high-pass filtered by the digital first-difference. Given that phase noise concentrates at frequencies well below the sampling frequency, the effects of aliasing are typically negligible. Therefore, the influence of phase noise in the performance of this kind of systems can be estimated calculating the result of high-pass filtering phase fluctuations.

**Figure 4:**
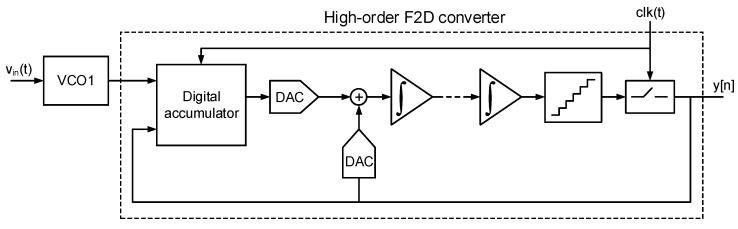
Generic high-order oscillator-based ΣΔ modulator.

However, this approach cannot be used to analyze other VCO-based modulators. For example, Figure 4 depicts a generic high-order VCO-based ADC composed of an oscillator and a high-order frequency to digital converter. The F2D converter may consist of a combination of analog integrators, oscillators, and digital circuitry [26,27,28,29]. These modulator topologies are specially interesting for sensor readout circuits because the sensor can be directly coupled to the first oscillator, similarly to the first-order modulator of Figure 1a. Unfortunately, given that these F2D converters are not based on the $1-z^{-1}$ differentiation, the phase noise generated in the first oscillator cannot be evaluated taking the approaches available in the literature.

This work presents a different approach to analyze the influence of phase noise in the performance of oscillator-based systems. Rather than calculating how oscillator phase noise (or jitter) affects the output spectrum of the system, we propose to calculate the input referred noise equivalent which can be directly compared to the input signal. This allows the calculation of the signal to noise ratio (SNR) of any oscillator-based system, regardless of the post-processing applied. We have taken as a case study the VCO-based ADC shown in Figure 1b for the sack of simplicity, but the analysis presented in this work can be applied to the system of Figure 1a as long as the relationship between the measurand and the oscillation frequency can be calculated.

In addition to phase noise, distortion may also limit the accuracy of the system for large input signals due to the nonlinear relationship between the input voltage and the oscillation frequency. This effect limits the dynamic range of the converter and plays an important role during the design of the VCO. As a main contribution, this paper describes a simulation methodology that can reduce the simulation time by orders of magnitude compared to noise enabled time domain simulations, yet keeping similar accuracy. This opens up the possibility to optimize the SNDR of oscillator-based systems by using iterative algorithms.

This paper is organized as follows. Section 2 reviews the phase noise of an autonomous oscillator and its basic properties. In Section 3, the input referred model of the phase noise is introduced and validated by simulation. Section 4 shows a comparison between the measurements of a 130 nm CMOS prototype and calculations carried out using the method proposed in the previous section. Section 5 describes how the input referred phase noise model and other calculations can be used to estimate the SNDR of a VCO-ADC without resorting to transient simulations. Finally, Section 6 concludes the paper.

## 2. Phase Noise of an Autonomous Oscillator

**Figure 5:**
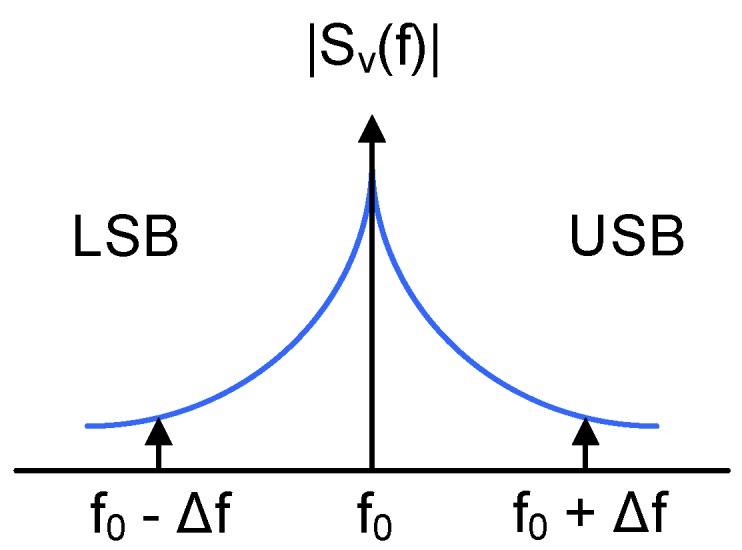
Spectrum of the oscillation around the center frequency.

Figure 5 depicts the power spectrum $S_{v}\left(f\right)$ of the output of an autonomous oscillator close to the center oscillation frequency ($f_{0}$). The electrical noise generated by the components that build up the oscillator is modulated by the oscillation and appears around the spectral components of the oscillation. From the correlation between the upper and the lower sidebands (USB and LSB) one can detect how the oscillation is perturbed: the noise at a given offset frequency (Δf) can modify the oscillation phase (Phase Modulation or PM), the amplitude (Amplitude Modulation or AM), or a combination of both [30,31]

In VCO-ADC applications, the oscillation is typically a square signal that can properly drive the digital circuitry that follows the VCO. This is done either by selecting a VCO topology which produces an square signal, or by passing a non-square oscillation through an amplitude limiter. In any case, amplitude noise is suppressed and the oscillator mainly exhibits PM noise, at least at the frequencies of interest. Our work is based on the assumption that $S_{v}\left(f\right)$ is dominated by phase fluctuations, either because of the oscillator topology, or because AM noise has been separated from PM noise.

According to the IEEE standard [32], the phase fluctuation is denoted by ϕ(t), and it is given in radians. The one-sided power spectral density (PSD) of the phase fluctuations is denoted by $S_{\phi }\left(\Delta f\right)$, and it is given in rad${}^{2}$/Hz. The phase noise of an oscillator is denoted by L(Δf) and it is defined in [32] as

(1) $$L\left(\Delta f\right)\equiv \frac{1}{2}S_{\phi }\left(\Delta f\right).$$

This is a redefinition of the historical formulation of L(Δf), which was defined as the PSD in one phase noise modulation sideband normalized to the fundamental tone power:

(2) $$L\left(\Delta f\right)=\frac{S_{v}\left(f_{0}+\Delta f\right)}{P_{\mathrm{carrier}}},$$

where $S_{v}\left(f_{0}+\Delta f\right)$ is the single sideband (SSB) PSD of the oscillation due to PM noise around $f_{0}$ (this is what a simple spectrum analyzer measures in the absence of AM noise). P_carrier_ is the total signal power around $f_{0}$, which is also equivalent to the power of the fundamental harmonic of the noiseless oscillation. Definitions (1) and (2) are approximately equivalent for low phase fluctuations, but they differ at low offset frequencies.

The PSD of the phase fluctuations was firstly described by David B. Leeson [17]. Phase fluctuations result from the combination of different noise types modulated by different mechanisms, what implies that $S_{\phi }\left(\Delta f\right)$ can be divided in several regions according to the dominant source and modulation [18,32].

**Figure 6:**
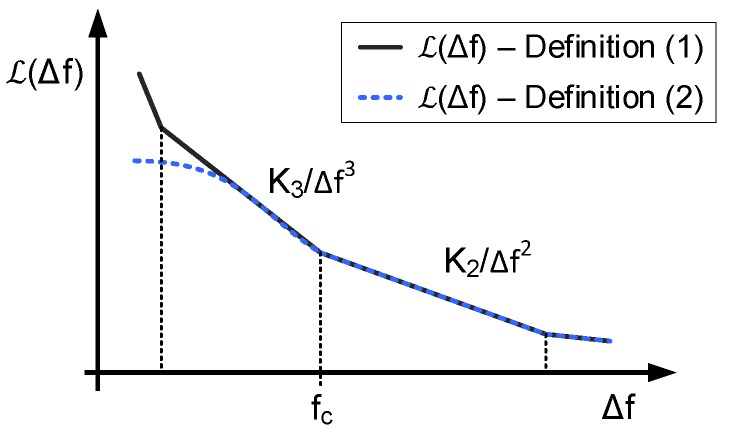
Simplified representation of L(f) commonly used at low and middle offset frequencies. Definitions (1) and (2) differ at very low offset frequencies.

$S_{\phi }\left(\Delta f\right)$ tends to infinity as Δf tends to zero. In the very low offset frequencies region, (1) and (2) are not compatible because it would mean that $S_{v}\left(f_{0}+\Delta f\right)$ also tends to infinity (what is senseless because signal power is finite). The spectrum of the oscillation close to the oscillation frequency has been discussed in [33,34,35,36], drawing the conclusion that the PSD tends to a constant finite value at very low offset frequencies, as illustrated in Figure 6. This graph depicts a simplified model of phase noise PSD on which two regions can be identified: $K_{3}/{\Delta f}^{3}$ describes the phase noise due to the FM modulation of flicker noise, whereas $K_{2}/{\Delta f}^{2}$ corresponds to the region dominated by white noise modulated in frequency. These two types of noise are typically dominant at middle frequencies, which is the band of interest of most applications. This simplified model allows the description of phase noise using only three parameters:

(3) $$L\left(\Delta f\right)=\frac{K_{3}}{\Delta f^{3}}+\frac{K_{2}}{\Delta f^{2}},$$

where $K_{3}$ and $K_{2}$ are parameters defined by noise levels. These two parameters are related by $K_{3}\;=\;K_{2}\;\cdot \;f_{c}$, where $f_{c}$ is the corner frequency which delimits the separation between the flicker noise and the white noise regions. For most of the oscillators used in VCO-ADCs, this description is accurate up to offset frequencies below the order of magnitude of the center oscillation frequency. Given that in most of applications this frequency is chosen to be well above the band of interest, this limit is relevant only for very high quality factor oscillators.

## 3. Input Referred Noise Model of a VCO

The oscillation frequency of a real VCO can be written as follows:

(4) $$f\left(t\right)=f_{0}\cdot \left(1+g\left(v_{in}\left(t\right)\right)\right)+f_{n}\left(t\right),$$

where f(t) is the oscillation frequency at instant *t*, $f_{n}\left(t\right)$ is the random oscillation frequency variation due to noise, and g(·) is a function that describes the relationship between the input signal $v_{in}\left(t\right)$ and the oscillation frequency. Function g(·) depends on the topology of the oscillator, but in most cases it is nonlinear and it can be linearized around $v_{in}\left(t\right)$ = 0 as follows:

(5) $$f\left(t\right)=f_{0}\cdot \left(1+k_{d}\cdot v_{in}\left(t\right)+\varepsilon \left(v_{in}\left(t\right)\right)\right)+f_{n}\left(t\right),$$

where $k_{d}$ is the relative frequency deviation factor (or gain) mentioned in Section 1, and $\varepsilon \left(v_{in}\left(t\right)\right)$ is a factor that represents the distortion components, which will be discussed in Section 5 and can be neglected in the noise analysis. $f_{n}\left(t\right)$ reduces the accuracy of the encoding process and limits the SNR of the converter because it is indistinguishable from a frequency variation produced by the input signal. In the same way as in conventional circuits the electrical noise is referred to the input, phase noise can be referred to the input of the VCO so it can be directly compared with the input signal, regardless the post-processing applied:

(6) $$f\left(t\right)=f_{0}\cdot \left(1+k_{d}\cdot \left(v_{in}\left(t\right)+r\left(t\right)\right)\right).$$

The signal r(t) is the Input Referred Phase Noise (IRPN) and represents a signal that, if applied to the input of a noiseless VCO, would produce an oscillation frequency variation similar to the one that a real oscillation exhibits with zero input due to phase noise.

**Figure 7:**
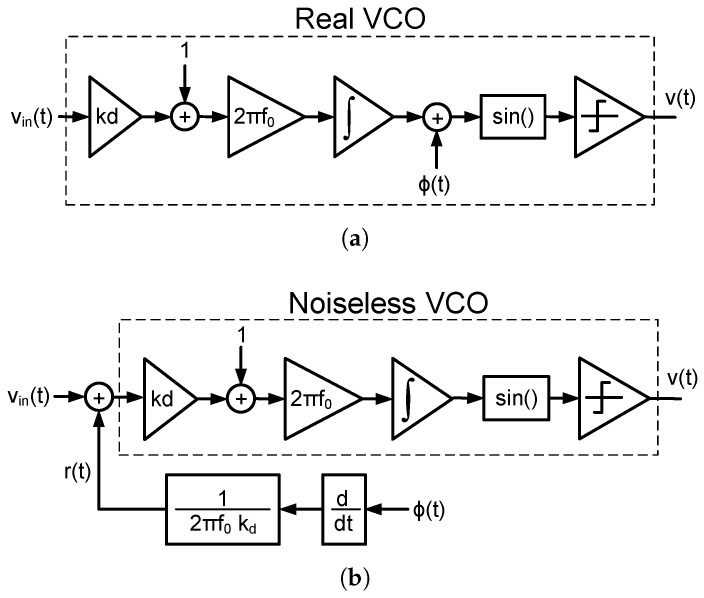
(**a**) Diagram of a real VCO with phase noise added to the phase of the oscillator; (**b**) Equivalent block diagram of the VCO with the phase noise referred to the input.

Figure 7a depicts the block diagram of a linear noisy VCO seen as a frequency integrator. In this model, the output of the integrator is the ideal phase of the oscillator to which the phase fluctuations are added. Among many others, one way to obtain a square wave from the phase is by calculating its sine and comparing the result with zero. Phase noise can be referred to the input of a noiseless VCO by simply multiplying the phase fluctuations ϕ(t) by the inverse of the transfer function seen from the input to the phase of the oscillator, as shown in Figure 7b. Therefore, the IRPN of a VCO can be expressed as

(7) $$r\left(t\right)=\frac{1}{2\pi k_{d}f_{0}}\cdot \frac{d\phi (t)}{dt}.$$

In addition, the one-sided PSD of the IRPN can be calculated as follows:

(8) $$S_{r}\left(\Delta f\right)=S_{\phi }\left(\Delta f\right){\left(\frac{2\pi \Delta f}{2\pi k_{d}f_{0}}\right)}^{2}.$$

Equation (8) can be combined with (1) to obtain

(9) $$S_{r}\left(\Delta f\right)=S_{\phi }\left(\Delta f\right)\frac{\Delta f^{2}}{k_{d}^{2}f_{0}^{2}}=L\left(\Delta f\right)\frac{2\Delta f^{2}}{k_{d}^{2}f_{0}^{2}}.$$

If the phase noise PSD follows the distribution described in (3) under the same assumptions, (9) can also be written as

(10) $$S_{r}\left(\Delta f\right)=\left(\frac{2K_{2}}{f_{c}f_{0}^{2}k_{d}^{2}}\right)\left(\frac{f_{c}}{\Delta f}+1\right).$$

**Figure 8:**
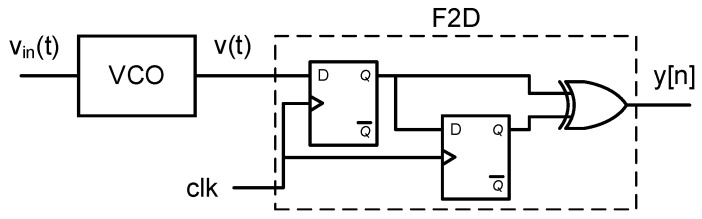
XOR-based VCO-ADC.

**Figure 9:**
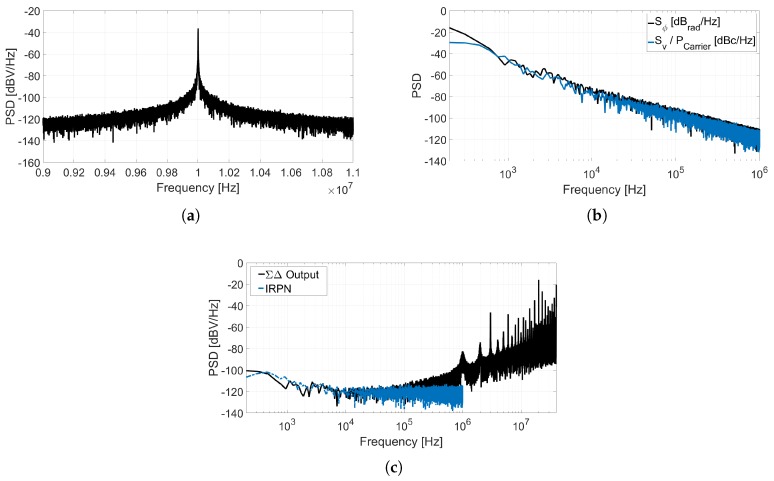
(**a**) Power spectrum of the oscillation $S_{v}\left(f\right)$; (**b**) Phase noise and phase fluctuation power spectral density; (**c**) IRPN and output data power spectral density.

The circuit depicted in Figure 8 is a first-order ΣΔ ADC composed by a VCO and an XOR-based F2D converter. Figure 9 shows the spectra of different nodes of this system obtained through a behavioral simulation including phase noise. Figure 9a describes the SSB PSD of the oscillation, $S_{v}\left(f\right)$, that can be used to estimate the phase noise by applying (2). Figure 9b illustrates the equivalence stated in (1), given that the $S_{\phi }\left(\Delta f\right)$ measured is about 3 dB above the L(Δf) estimated. Figure 9c compares the IRPN calculated by applying (9) to phase noise, to the spectrum of the data converter output bitstream y[n] divided by the ADC gain (so it is also referred to the input). It can be observed that the matching between both simulations is limited up to the frequency on which quantization noise exceeds phase noise. The gain of this XOR-based VCO-ADC can be derived from the term *BB* introduced in [16]. At frequencies well below the sampling frequency, it can be demonstrated that the gain of this ADC is

(11) $$BB\left(f\right)\approx \frac{2k_{d}f_{0}}{f_{s}},\;\mathrm{if}\;f\ll f_{s}.$$

The SNR is given by the ratio between the signal power and noise power. Assuming that the effects of aliasing are negligible, input referred noise power can be calculated integrating the IRPN described in (9) between the limits of the band of interest. The SNR of the VCO-ADC due to phase noise can be calculated comparing the input signal power to the input referred noise power as follows:

(12) $$SNR=10\cdot log_{10}\left(\frac{P_{s}ignal}{P_{n}oise}\right)=10\cdot log_{10}\left(\frac{x_{peak}^{2}/2}{\int_{f_{Lo}}^{f_{Hi}}S_{r}\left(\Delta f\right)d\Delta f}\right),$$

where $f_{Lo}$ and $f_{Hi}$ are respectively the lower and upper limits of the band of interest, and $x_{peak}$ is the amplitude of the input tone.

## 4. Prototype Measurements

**Figure 10:**
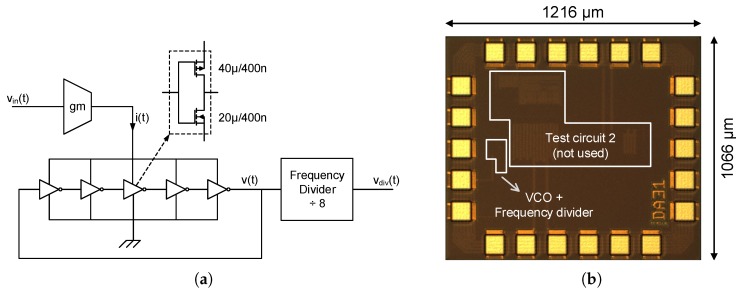
130 nm CMOS prototype description. (**a**) Circuit; (**b**) Die micrograph.

A VCO fabricated in 130 nm standard CMOS technology has been measured to check the accuracy of the calculations proposed in the previous section. The oscillator prototyped is a 5-stage Ring Oscillator driven by the current provided by a single PMOS which works as a transconductor, as shown in Figure 10. This architecture has been chosen due to its simplicity and acceptable performance, taking into account that the purpose of this test is not the design of a high-performance oscillator but the validation of the equations proposed in this work. A frequency divider has been used to reduce the oscillation frequency by a factor of 8 and thus overcome pad limitations. The rest of the subcircuits marked in Figure 10b have not been used in this test, and the output of the VCO has been sampled and post-processed in MATLAB^®^ emulating the behavior ot the XOR-based F2D converter shown in Figure 8.

**Figure 11:**
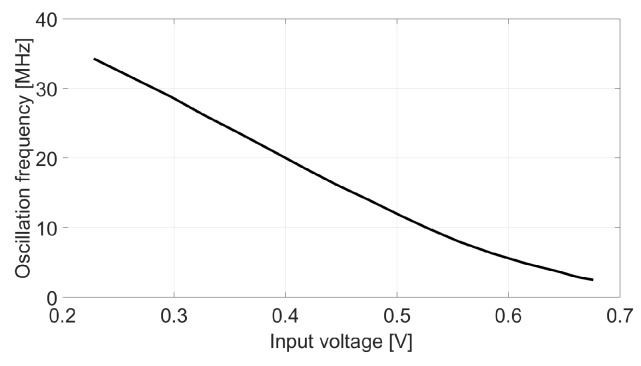
Measured oscillation frequency vs. input voltage.

First, the oscillation frequency response has been characterized by a DC sweep at the VCO input, as shown in Figure 11. The nominal frequency of the oscillator is 12 MHz (96 MHz before the divider), which is reached when the input signal is 500 mV. The gain of the VCO can be obtained from this plot by calculating the slope of this graph around the point ($v_{in}$ = 500 mV, $f_{0}$ = 12 MHz). In this case the absolute value of the slope is about 76.4 MHz/V, what leads to $k_{d}$ = 6.4 V${}^{-1}$.

**Figure 12:**
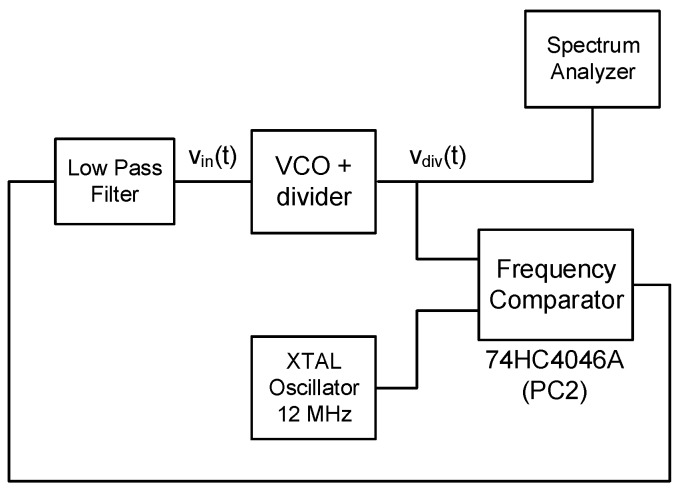
Test fixture for phase noise measurements.

After this, the oscillator has been connected to a frequency stabilization loop, as shown in Figure 12. We have used the phase and frequency comparator available in the commercial integrated circuit 74HC4046A (*PC2* output). This loop, whose bandwidth is well below the band of interest, keeps the oscillation frequency centered at 12 MHz compensating any undesired slow frequency drift, and enabling more accurate measurements of $S_{v}\left(f\right)$ with a spectrum analyzer.

**Figure 13:**
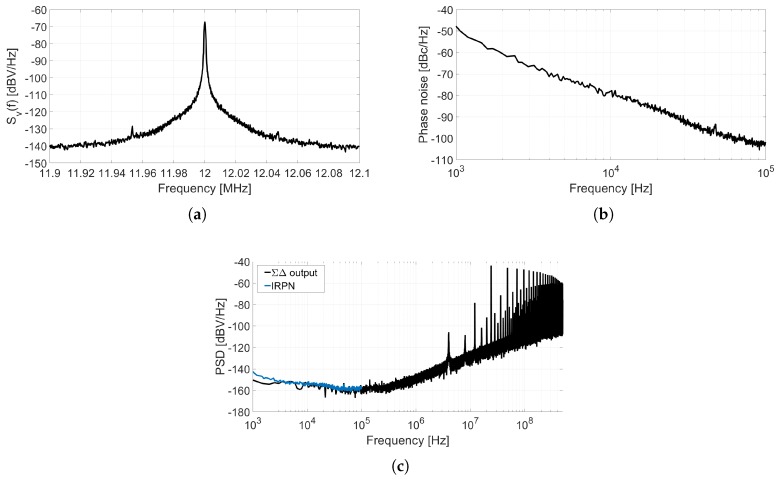
(**a**) $S_{v}\left(f\right)$ measured with an spectrum analyzer; (**b**) Phase noise derived from $S_{v}\left(f\right)$; (**c**) Comparison between the IRPN calculated from L(Δf) and the DFT of the measured ADC output.

This test fixture has been used to measure and calculate the graphs presented in Figure 13. Figure 13a shows the spectrum of the oscillation around $f_{0}$, which has been used to estimate the phase noise depicted in Figure 13b by applying Equation (2). Input referred phase noise is calculated by applying Equation (9) to the phase noise shown in Figure 13b. The result is presented in Figure 13c together with the PSD of the output of the ADC properly scaled by the inverse of the ADC gain.

Equation (12) can be used to calculate the SNR due to phase noise. We take as example a 0.55 ${\mathrm{mV}}_{\mathrm{peak}}$ input tone and a band of interest from 5 kHz to 50 kHz. The noise power obtained by numerically integrating $S_{r}\left(\Delta f\right)$ across this band is 11.6 μV${}^{2}$, and therefore the SNR predicted is 41.16 dB.

**Figure 14:**
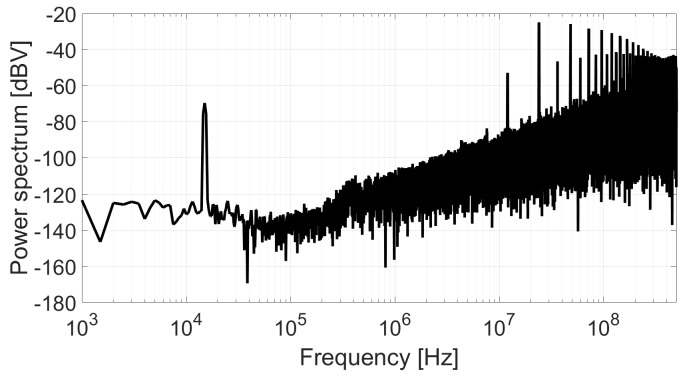
Power spectrum of the ADC output.

After the idle channel measurement, the test fixture has been modified adding a balun transformer at the VCO input in order to inject a modulating signal in the loop without modifying the conditions used in the previous measurement. A tone of 0.55 ${\mathrm{mV}}_{\mathrm{peak}}$ at 15 kHz has been added to the input of the VCO through a balun transformer. Figure 14 shows the power spectrum of the converter after applying the same post-processing than in the previous measurement. The SNR obtained from this test is 42.72 dB in the bandwidth from 5 kHz to 50 kHz. Therefore, the deviation between theory and simulation is less than 2 dB.

## 5. VCO Simulation and SNDR Estimation

Due to the time-varying behavior of oscillators, classical analysis based on small-signal linearization such as AC and Noise analysis are not suitable for simulating VCOs. Transient noise analysis can accurately simulate the behavior of the VCO-based system, but this demands a significant amount of computing power and time. This issue is magnified in some VCO applications on which the time constants of the oscillator subcircuits are several orders of magnitude shorter than the length of the simulation required to obtain relevant results. Given the highly iterative nature of the design and optimization processes, transient simulations are not always an efficient tool to face the design phase.

In this section, we describe how to estimate the limitations that a given VCO imposes to a VCO-based system in terms of distortion and phase noise without performing long transient simulations. Some simulation options may differ from the ones used in this section depending on the design environment (as a reference, in this case we are using Cadence^®^ Virtuoso^®^ Design Environment version IC6.1.6.500.6). The model proposed in Section 3 and validated in Section 4 is specially useful to characterize the performance of the VCO in terms of phase noise. At the end of this section we also show some simple calculations that allow to estimate the distortion of the oscillator.

**Figure 15:**
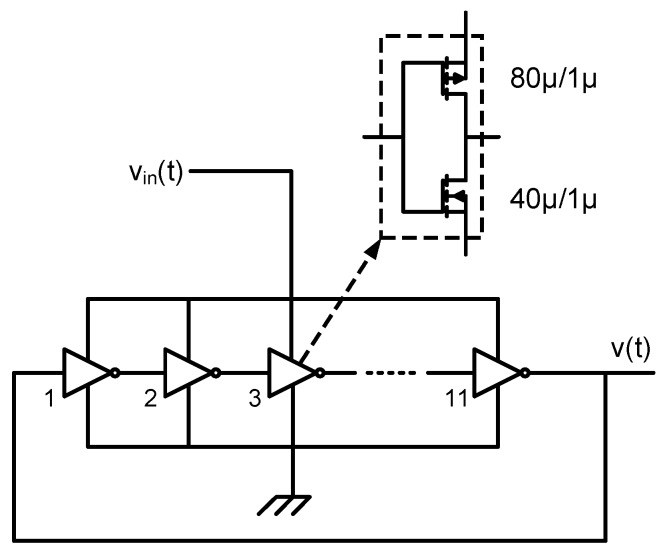
Simulated voltage-controlled ring oscillator.

The voltage-controlled ring oscillator (VCRO) shown in Figure 15 has been taken as a case study assuming a bandwidth from 1 kHz to 100 kHz. The limits of this oscillator can be analyzed by performing a transient simulation of the VCO connected to a F2D converter. The XOR-based circuit of Figure 8 can be used for this purpose, in combination with a sampling frequency high enough to set the quantization noise below the phase noise in the band of interest. Alternatively, we can sample the oscillation very fast and emulate the F2D a posteriori, what would save the computational effort of simulating the F2D. In any case, simulating this VCO whose center oscillation frequency is about 60 MHz with the appropriate settings to obtain an acceptable accuracy may take a few hours. In our case, with the simulation setup that we have available, simulating this circuit for 4 milliseconds takes between 3 and 16 h, depending on the maximum step size chosen.

There are other tools capable of simulating the behavior of oscillators and their noise. For example, Cadence^®^ Spectre^®^ RF Option provides the Harmonic Balance (HB) analysis and the Shooting Newton method to calculate the periodic steady-state (PSS) of oscillators. The Shooting Newton method calculates the time-domain PSS and it is suitable for highly nonlinear circuits such as ring oscillators, relaxation oscillators, and frequency dividers. HB performs a frequency-domain analysis, which is more efficient for weak and midly nonlinear circuits such as LC oscillators [37]. The VCRO simulated is a strongly nonlinear circuit with sharp transitions, so the Shooting Newton method is in principle more suitable. A PSS simulation can determine in a few seconds that the oscillation frequency of this VCO is $f_{0}$ = 60.57 MHz. Taking advantage of the PSS sweep tool, we can perform several PSS analysis while sweeping the input voltage in order to calculate the VCO sensitivity, which in this case is $k_{d}$ = 2.28 V${}^{-1}$.

**Figure 16:**
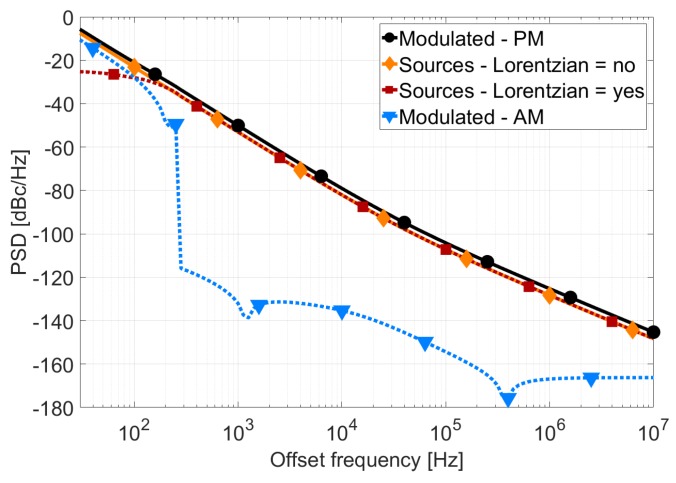
Comparison between different periodic noise simulations.

After calculating the PSS, the pnoise analysis can be used to estimate the phase noise of the oscillator. Table 1 shows the type of noise that is calculated for different simulation setups. Figure 16 shows the results of pnoise simulations with the different options described in the table. On one hand, from the “Modulated” noise type it can be observed that the AM noise is negligible compared with the PM noise for most of the frequencies. On the other hand, phase noise computed by “Sources” noise type is 3 dB below the PM noise and it is limited at low offset frequencies if “lorentzian = yes”, in concordance with (1).

In addition to phase noise, the distortion of the VCO is an important nonideality that can limit the performance of the ADC [24,38]. Distortion is due to the nonlinear relationship between the input magnitude and the oscillation frequency, which corresponds to the function g(·) introduced in (4) and can be expressed as the following polynomial:

(13) $$g\left(v_{in}\left(t\right)\right)\approx k_{d1}v_{in}\left(t\right)+k_{d2}v_{in}{\left(t\right)}^{2}+k_{d3}v_{in}{\left(t\right)}^{3}+\ldots$$

For a sinusoidal input with amplitude *A* and frequency $\omega_{x}$, the oscillation frequency can be rewritten as follows:

(14) $$\begin{array}{cc} f(t)= & f_{0}\cdot (1+k_{d1}A\cdot \cos\left(\omega_{x}t\right)+k_{d2}A^{2}\cdot \cos^{2}\left(\omega_{x}t\right)+\\ & k_{d3}A^{3}\cdot \cos^{3}\left(\omega_{x}t\right)+k_{d4}A^{4}\cdot \cos^{4}\left(\omega_{x}t\right)+\ldots ), \end{array}$$

which after a few trigonometrical transformations can be expressed as:

(15) $$\begin{array}{cc} f(t)= & f_{0}\cdot (\left(1+\frac{A^{2}k_{d2}}{2}+\frac{3A^{4}k_{d4}}{8}+\frac{10A^{6}k_{d6}}{32}+\ldots \right)+\\ & \cos\left(\omega_{x}t\right)\left(Ak_{d1}+\frac{3A^{3}k_{d3}}{4}+\frac{10A^{5}k_{d5}}{16}+\ldots \right)+\\ & \cos\left(2\omega_{x}t\right)\left(\frac{A^{2}k_{d2}}{2}+\frac{A^{4}k_{d4}}{2}+\frac{15A^{6}k_{d6}}{32}+\ldots \right)+\\ & \cos\left(3\omega_{x}t\right)\left(\frac{A^{3}k_{d3}}{4}+\frac{5A^{5}k_{d5}}{16}+\ldots \right)+\ldots ). \end{array}$$

The amount of terms required to accurately calculate the signal to distortion ratio (SDR) depends on the oscillator topology and on the application, but in most of the cases {$|Ak_{d1}|$, $|A^{2}k_{d2}|$, $|A^{3}k_{d3}|$} ≫ {$|A^{4}k_{d4}|$, $|A^{5}k_{d5}|$, … }. Therefore, the signal-to-distortion ratio can be estimated as follows:

(16) $$\mathrm{SDR}\left(A\right)\approx 10\log_{10}\left(\frac{{\left(4Ak_{d1}\right)}^{2}}{{\left(2A^{2}k_{d2}\right)}^{2}+{\left(A^{3}k_{d3}\right)}^{2}}\right).$$

**Figure 17:**
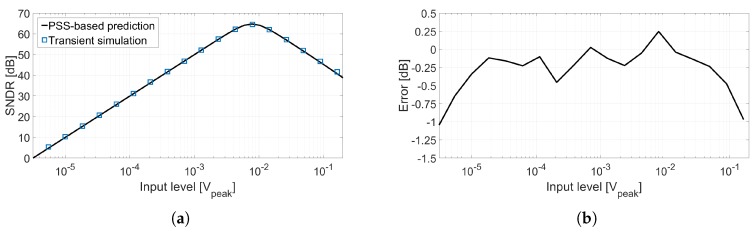
(**a**) Performance of circuit shown in Figure 15 calculated using transient simulations and estimated from PSS and Pnoise simulations; (**b**) SNDR difference between both methodologies.

Figure 17a shows the comparison of the dynamic range plot of the VCO-ADC shown in Figure 15 calculated using two methods: the set of blue square markers represents the result of transient simulations; the black curve is the result of applying the equations proposed in this work to the PSS-based simulations of the circuit. The SDR has been estimated computing a PSS-Sweep to obtain the oscillation frequency versus input voltage curve (i.e., the $g(v_{in})$ function), from which coefficients $k_{d1}$, $k_{d2}$, and $k_{d3}$ can be obtained through polynomial curve fitting. On the other hand, the SNR has been calculated applying Equations (9) and (12) to the result of a pnoise simulation. The difference between both sets of simulations is shown in Figure 17b. It can be observed that the SNDR obtained following both methodologies is similar for most of the input amplitudes, although for very large signals our polynomial approximation seems to have some limitations in comparison with transient simulations. However, transient simulations require several hours while the SNDR estimation based on PSS simulations only takes a few minutes. This computation time reduction is a major advantage because it enables the use of the estimation in sweeps, sensitivity analysis, and iterative optimization processes.

## 6. Conclusions

In this work we have proposed a methodology to evaluate the performance of oscillator-based sensor readout circuits, which are typically limited by phase noise and distortion. Our estimations are based on two simulations: a PSS sweep is used to calculate the oscillation frequency, the gain, and the distortion of the oscillator; and a periodic noise analysis is used to calculate the phase noise, which can be subsequently referred to the input of the converter to estimate the SNR. A 130 nm CMOS prototype has been fabricated to check the validity of our SNR estimation model. The difference between the estimation of our theoretical model and computer simulations differ from practical measurements in less than 2 dB. This paper proposes a new simulation strategy that allows to estimate the SNDR of oscillator-based systems avoiding transient simulations. As an advantage, the computing time of our proposed method is at least one order of magnitude faster than an equivalent transient simulation and provides similar results within 2 dB of accuracy. This precision and speed permits an interactive optimization of the oscillator circuit.

## Figures and Tables

**Table 1:** Simulation results for different setups.

Pnoise Setup	Simulation Result
Modulated-PM	$S_{\phi }\left(\Delta f\right)$
Sources-Lorentzian = no	L(Δf)-Definition (1)
Sources-Lorentzian = yes	L(Δf)-Definition (2)
Modulated - AM	Amplitude fluctuations
